# Female Reproductive System Neoplasms in Neotropical Primates

**DOI:** 10.1111/jmp.70027

**Published:** 2025-06-25

**Authors:** Asheley H. B. Pereira, Fernanda C. Rocha, Marcos P. S. Carvalho, Bruna E. P. Barbosa, Daniel A. Balthazar, Silvia B. Moreira, Alcides Pissinatti, Daniel G. Ubiali

**Affiliations:** ^1^ Pathological Anatomy Sector (SAP), Department of Epidemiology and Public Health, Veterinary Institute Federal Rural University of Rio de Janeiro Seropédica RJ Brazil; ^2^ BioParque Do Rio, Quinta da Boa Vista São Cristóvão RJ Brazil; ^3^ Facultat de Veterinària, Universitat Autònoma de Barcelona Barcelona Spain; ^4^ Departament of Veterinary Medicine and Surgery Veterinary Institute, UFRRJ Seropédica Brazil; ^5^ Rio de Janeiro Primatology Center (CPRJ/INEA) Guapimirim Brazil

**Keywords:** female reproductive health, ovarian neoplasms, uterine neoplasms

## Abstract

**Background:**

There are no previous descriptions of the prevalence of spontaneous female reproductive neoplasms in neotropical primates.

**Methods:**

A 6‐year study of pathological records from the Anatomy Pathology Sector from the Federal Rural University of Rio de Janeiro (SAP/UFRuralRJ) was conducted. All cases of female nonhuman primate taxa belonging to the Platyrrhini parvorder with primary reproductive neoplasms were studied.

**Results:**

The overall prevalence of female reproductive neoplasms in neotropical primates from 2019 to 2024 was 2.6% (6/228). Ovarian neoplasms were all classified as lesions without clinical significance. Uterine adenocarcinomas were observed in 50% (3/6) and were considered the cause of death in all cases.

**Conclusions:**

Our findings suggest that there are no significant differences in the prevalence of ovarian and uterine spontaneous neoplasm presentation in neotropical primates. Despite the low prevalence, uterine adenocarcinomas should be included as a potential cause of neotropical primate deaths.

## 1 Introduction

Knowledge of nonhuman primate reproductive health is essential for medical and conservation sciences. The number of species and subspecies recognized as comprising the Platyrrhini parvorder, the neotropical primates, has increased considerably since the early 20th century. Currently, 218 different taxa are grouped into 24 genera and five families [1].

Several studies have described the anatomical and physiological features of the reproductive system in different neotropical primate species [2, 3, 4, 5, 6]. Herein, we describe the epidemiological and clinicopathological features of spontaneous female reproductive neoplasms in neotropical nonhuman primates.

## 2 Materials and Methods

The diagnostic reports from the Anatomy Pathology Sector of Federal University Rural of Rio de Janeiro (SAP/UFRuralRJ) from January 2019 to December 2024 were reviewed. All cases of female nonhuman primate taxa belonging to the Platyrrhini parvorder with primary reproductive neoplasms were incorporated into this study. The epidemiological data regarding sex and age in each case were obtained from the technical veterinarian of the colonies. All procedures were conducted in full compliance and approval by the Brazilian Ministry of the Environment (SISBIO 30939‐12). All neoplasm cases were categorized according to the 2020 WHO classification criteria for female genital tumors [7].

### 2.1 Necropsy, Histopathology, and Immunohistochemistry (IHC)

In the cases of death or euthanasia in nonhuman primates, the standardized necropsies were performed by two veterinary pathologists of the SAP/UFRuralRJ or by the colony technical responsible. During the procedure, personal protective equipment was used. A complete set of tissue samples was collected from each primate and fixed in a 10% buffered formalin solution. The tissue samples stored in formalin were fixed and submitted to routine histological processing. Histological slides were stained with Hematoxylin and Eosin and then observed under optical microscopy.

Tissue blocks were sectioned into 3‐μm‐thick slices and mounted onto silane‐coated microscope slides. All cases of neoplastic proliferations were subjected to IHC according to the manufacturer's recommendations, with minor modifications, using anticytokeratin‐7 (Dako, Clone 7, dilution 1:200). Antigen retrieval was achieved using Tris‐EDTA pH 9.0 (Dako Cytomation, www.agilent.com/en/dako‐products) at 96°C for 30 min. The sections were then incubated in methanol: H_2_O_2_ solution (97%:3%) for 20 min to block nonspecific binding. An endometrioid carcinoma of a hybrid *Leontopithecus* sp. was used as a positive control [8]. For negative controls, phosphate‐buffered saline (PBS) replaced the primary antibody. Peroxidase activity was visualized with EnVision FLEX DAB Chromogen System (Dako Cytomation, www.agilent.com/en/dako‐products). The sections were counterstained with hematoxylin, coverslipped, and viewed with an optical microscope.

## 3 Results

From January 2019 to December 2024, we performed 228 necropsies and histopathological examinations on female Platyrrhini nonhuman primates at the SAP/UFRuralRJ. The overall prevalence of female reproductive neoplasms in neotropical primates during this study period was 2.6% (6/228). Out of researched Pitheciidae cases, 66.6% (4/6) were from the Rio de Janeiro Primatology Center (CPRJ), Guapimirim, RJ, 16.7% (1/6) from the BioParque do Rio, Rio de Janeiro, RJ, and 16.7% (1/6) from the Triage Center of Wild Animals (CETAS), Seropédica, RJ. The responsible veterinarians declared that no contraceptive drugs were used in any of these colonies. Although most cases were observed in callitrichids (3/6 cases), the highest prevalence was noted in the Pitheciidae family, with 5.9% (1/17) of the cases, followed by Atelidae with 5% (1/20), Cebidae with 2.8% (1/35), and Callitrichidae with 2% (3/154) of the cases. Granulosa cell tumors are the most common ovarian neoplasms in neotropical primates, and all ovarian‐derived neoplasms were classified as lesions without clinical significance. Uterine neoplastic proliferations were observed in 50% (3/6) of individuals. All uterine neoplasms were adenocarcinomas and were considered the cause of death in all cases. The species, sex, age, and histogenesis of each case are presented in Table 1.

**TABLE 1:** Spontaneous Female Reproductive Neoplasms in Neotropical Primates.

No	Species	Family	Age^a^	Location	Neoplasm	Metastasis
1	*Alouatta seniculus*	Atelidae	17	Right ovary	Granulosa cell tumor	—
2	*Sapajus* sp.	Cebidae	Adult	Left ovary	Granulosa cell tumor	—
3	*Callimico goeldii*	Callitrichidae	7	Both ovaries	Micropapillary borderline serous tumor	—
4	*Leontopithecus chrysopygus*	Callitrichidae	3	Uterus	Uterine adenocarcinoma	—
5	*Calicebus personatus*	Pitheciidae	6	Uterus	Uterine adenocarcinoma	Peritoneum
6	*Leontopithecus* sp.	Callitrichidae	19	Uterus	Endometriod carcinoma	Urinary bladder Small intestine, jejunum Large intestine, rectum

^a^ Years.

### 3.1 Ovarian Neoplasms

An *Alouatta seniculus* was submitted for veterinary clinical care due to anorexia, apathy, and prostration. Despite therapeutic efforts, the primate died and was submitted for necropsy. Macroscopically, the right ovary was diffusely and moderately enlarged, firm, and multilobulated, with measurements of 2 x 1.5 x 1 cm. There were well‐demarcated, regular, and yellow‐to‐brown millimetric nodules on the cut surface. Histologically, the right ovary parenchyma was effaced and compressed by a well‐demarcated, densely cellular neoplasm composed of well‐differentiated granulosa cells arranged in small islands and cords and supported by a thin fibrovascular stroma. The neoplastic cells showed indistinct cell borders, a scant amount of pale eosinophilic cytoplasm, and a round to ovoid nucleus with finely stippled chromatin and an indistinct nucleolus. Cellular pleomorphism was mild, and there was mild anisocytosis and anisokaryosis. One mitotic figure was observed per high‐power field (2.37 mm^2^, Obj. 40x).

**FIGURE 1:**
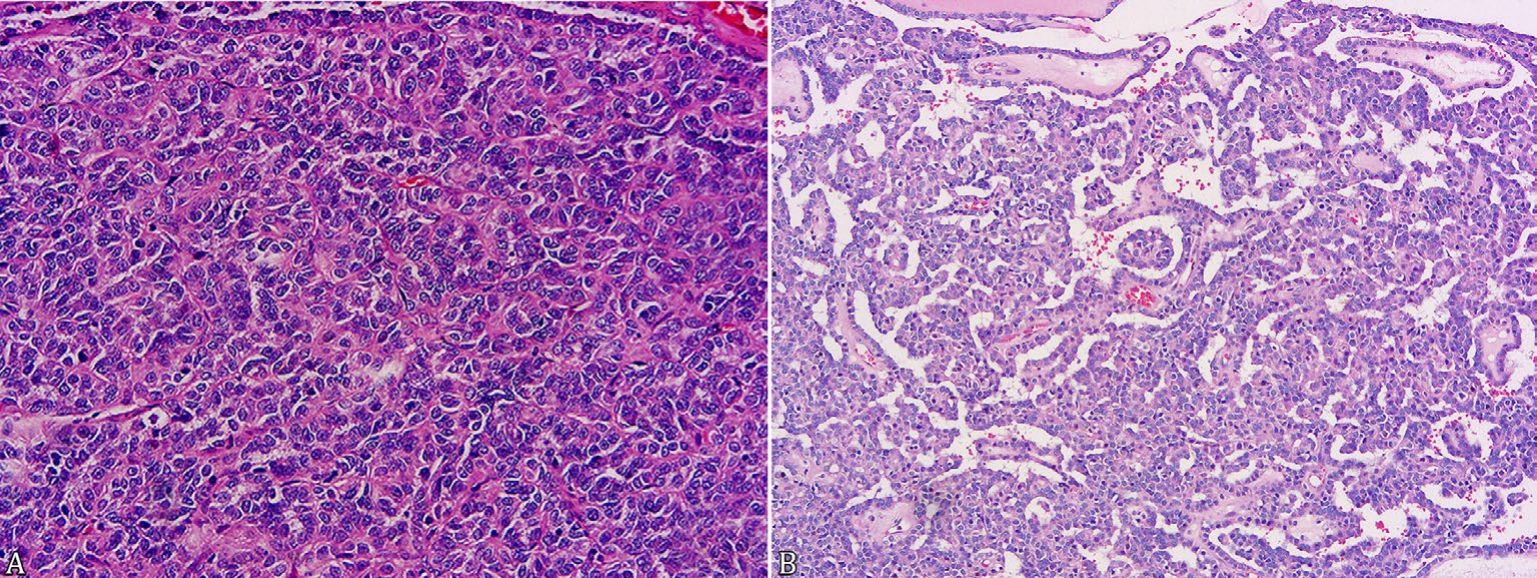
Histopathology of ovarian neoplasm in neotropical primates. (A) Granulosa cell tumor in a *Sapajus* sp.: Well‐differentiated granulosa cells are arranged in nests and supported by a thin fibrovascular stroma (HE, Obj. 40x) (B) Borderline micropapillary serous tumor in a *Callimico goeldii* : Epithelial cells are arranged in multiple papillae with extensive and complex branching, supported by a fibrous stroma (HE, Obj. 20x).

A *Sapajus* sp., without evident clinical signs, presented an increase in volume in the left ovary during an elective contraceptive castration procedure. Grossly, a unilateral increase in volume was observed in the left ovary, measuring 1.5 x 1 x 0.5 cm. The left ovary was diffusely firm, slightly multilobulated, and brown. On the cut section, well‐demarcated multifocal to coalescent nodular areas were observed. After the surgical procedure, both ovaries and uterine tubes were submitted for histopathology. Histologically, the normal ovarian architecture was effaced and partially replaced by an encapsulated, well‐differentiated, moderately cellular neoplasm. The neoplasm was composed of polyhedral neoplastic cells arranged in small nests, sheets, and sometimes with a follicular pattern supported by a thin fibrovascular stroma. Neoplastic cells have homogeneous and scarce eosinophilic cytoplasm with very distinct cell borders. The nuclei are round to oval, with granular chromatin and a distinct nucleolus. Cellular and nuclear pleomorphism was mild. Four mitoses were observed in 10 high‐power fields (2.37 mm^2^, Obj. 40x). A diagnosis of granulosa cell tumor was made based on the morphological features (Figure 1A). There are no morphological changes in the right ovary and uterine tubes.

A *Callimico goeldii* with marked distension and abdominal pain died after presenting dystocia. Both ovaries exhibited irregular surfaces on necropsy, with multiple millimetric cystic areas filled with a discreet amount of serous, translucid fluid. Histologically, a moderately cellular neoplasm originating from the surface ovarian epithelium and extending especially into the tunica albuginea and subjacent ovarian parenchyma was observed. The neoplasm was composed predominantly of cuboidal cells arranged in multiple papillae with extensive and complex branching supported by a fibrous stroma. Cilia are frequently observed at the apical end of epithelial cells. The epithelial cells of the papillae are clustered and form stratified areas composed of three or more cell layers, exhibiting a moderately eosinophilic cytoplasm, a central nucleus, sometimes with finely stippled chromatin. Nuclear atypia was mild to moderate, and four mitotic figures were observed in 10 high‐power fields (2.37 mm^2^, Obj. 40x). Multifocal areas of varying sizes, with a large amount of amorphous eosinophilic material, were associated with rare pyknotic debris and necrosis. A borderline micropapillary serous tumor diagnosis was made based on the morphological features (Figure 1B).

### 3.2 Uterine Neoplasms

**FIGURE 2:**
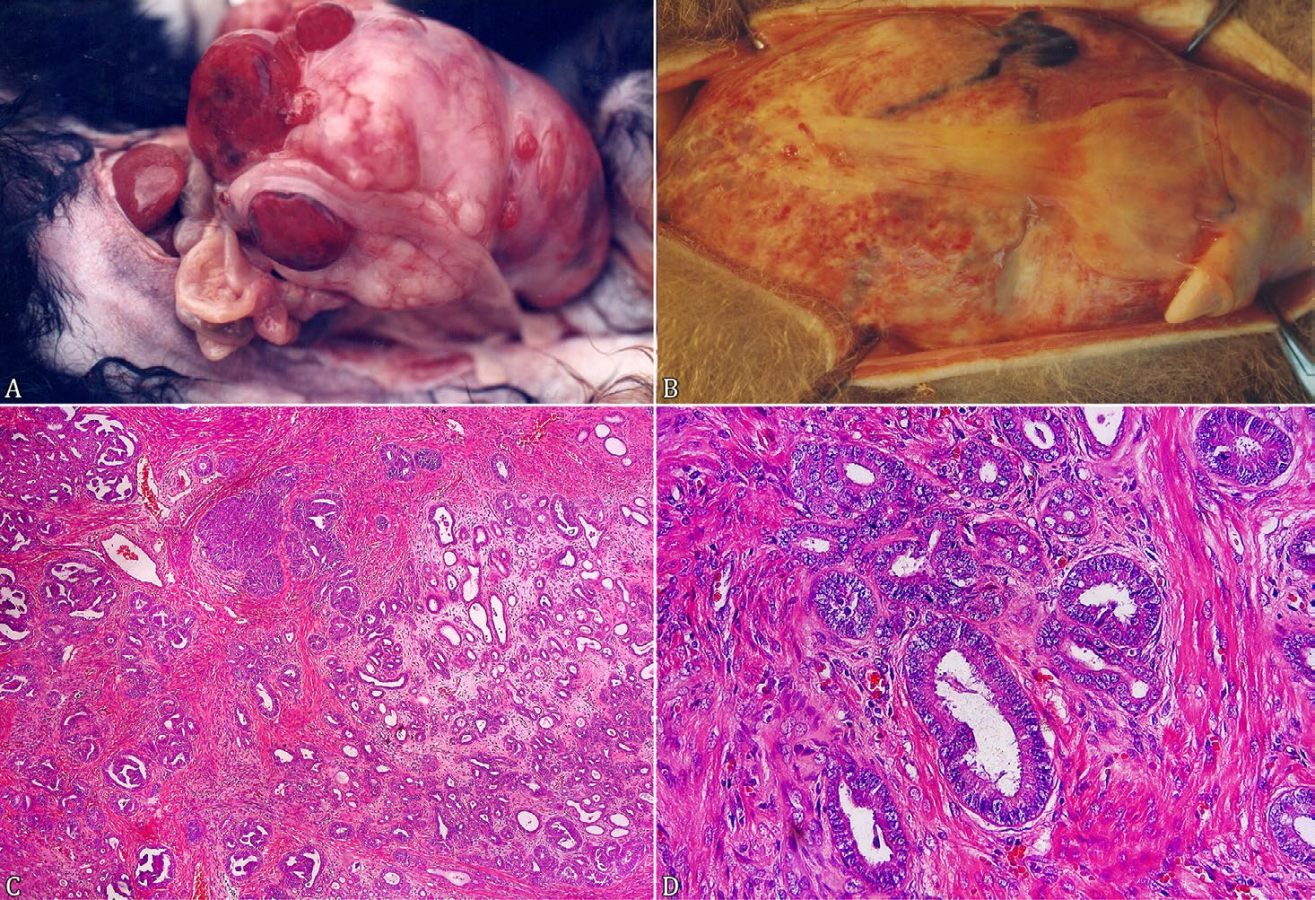
Gross and histopathology of uterine neoplasm in neotropical primates. (A) *Leontopithecus chrysopygus* had an irregular and increased uterus with multiple friable, red‐dark, variable‐sized nodules on its surface. (B) *Callicebus personatus* with a markedly enlarged and irregular uterus. (C) Moderately differentiated uterine adenocarcinoma in a *C. personatus* (HE, Obj. 5x) (D) Uterine neoplastic proliferation composed of moderately differentiated tall to polyhedral epithelial cells arranged in tubules and small nests in a *C. personatus* (HE, Obj. 40x) (Figure 2C,D).

A specimen of *Leontopithecus chrysopygus* was found dead in the enclosure without a clinical history. On necropsy, there was marked abdominal distension and a diffusely irregular and multinodular uterus measuring 8 x 6 x 4 cm. Multiple friable, dark redvariable‐sized nodules were present on the uterine surface (Figure 2A). The nodules were infiltrative, nonencapsulated, and irregular on the cut surface. The abdominal cavity showed a moderate amount of dark red, opaque fluid. Histologically there was no encapsulated and densely cellular neoplasm composed of tall columnar cells arranged mainly in papillae and supported by a fibrovascular and myxoid stroma, which was effacing and expanding the endometrium and infiltrating the subjacent myometrium. The neoplastic cells were large, sometimes polyhedral, with moderate eosinophilic cytoplasm and poorly defined borders. The nuclei were large and oval, with finely stippled chromatin and an evident nucleolus. The pleomorphism was moderate, with occasional karyomegalic cells, and eight mitotic figures were observed in 10 high‐power fields (2.37 mm^2^, Obj. 40x). The neoplastic cells exhibited strong cytoplasmic immunolabeling for anticytokeratin 7. A uterine adenocarcinoma diagnosis was made based on the morphological and immunohistochemical findings.

A specimen of *Callicebus personatus* was found dead without clinical history. On necropsy, the abdominal cavity was markedly distended by an irregular and enlarged uterus with multiple nondemarcated, multilobulated, firm nodules (Figure 2B). The peritoneum had a friable, dark‐red, irregular nodule measuring 2 x 2 x 1 cm. On histology, the uterine architecture was replaced by an infiltrative, densely cellular neoplastic proliferation composed of moderately differentiated tall to polyhedral epithelial cells arranged in tubules and small nests (Figure 2C,D). There was moderate anisocytosis and anisokaryosis, and 10 mitotic figures were observed in 10 high‐power fields (2.37 mm^2^, Obj. 40x). The peritoneal nodule showed neoplastic cellular proliferation similar to that observed in the uterine section and was considered a metastasis. The IHC revealed diffuse and strong immunolabeling against anticytokeratin 7. A poorly differentiated uterine adenocarcinoma diagnosis was made based on the pathological and immunohistochemical features.

A hybrid *Leontopithecus* sp. with vaginal bleeding history died due to a uterine endometrioid carcinoma with metastasis in the in the urinary bladder, jejunum, and rectum. The clinicopathological data were previously reported [8].

## 4 Discussion

Herein, we present a 2.6% (6/228) prevalence of spontaneous reproductive female neoplasms in neotropical primates based on a routine diagnostic approach. It was previously believed that nonhuman primates rarely develop neoplasms of the female reproductive system [9]. A translational study of our research group revealed 3.8% (1/26) cases of uterine carcinoma in the neotropical primates from the Pitheciidae family. However, descriptions of the prevalence and incidence of reproductive neoplasms are increasingly common in colonies of Old World Monkeys (OWM) and apes [10, 11, 12, 13, 14]. The overall prevalence in neotropical primates was lower than the 7%–15% rate reported for OWM [15]. This fact can probably be attributed to the typical presentation of uterine leiomyomas in OWM, which was not seen in this study. Our findings suggest that there are no significant differences in the prevalence of ovarian and uterine neoplasm presentation in neotropical primates. The reason for this difference is uncertain, but the spectrum and presentation of spontaneous lesions can be influenced by genetics and may vary significantly from colony to colony [16].

Ovarian tumors are a common finding in aging macaques, apes, and women and most commonly arise from either the epithelial or follicular structures within the ovary [13, 17]. As seen in macaques [9, 13, 17], the most common ovarian neoplasm in the neotropical primates of this study was the granulosa cell tumor. These tumors are typically benign and are considered, in most cases, to be lesions of no clinical significance [18]. In women, ovarian neoplasms are primarily derived from the surface epithelium, and the ovarian serous borderline tumor is the most common type of borderline tumor arising in the ovary [19]. In contrast, surface epithelial ovarian tumors are less common in nonhuman primates [20]. The *C. goeldii* case presented here shares the same morphological aspects as those described in humans [19]. It should be considered a differential diagnosis for ovarian neoplasia in neotropical primates.

Malignant neoplasia of the reproductive system represents nearly 40% of all cancers affecting women worldwide [21]. Ad enocarcinomas derived from the endometrium are considered rare in nonhuman primate species [22]. All cases of uterine neoplastic proliferations in this study were adenocarcinomas directly related to the individual's death. Based on this fact, uterine adenocarcinomas in neotropical primates have a poor prognosis and potential metastatic spread. The diagnosis was made in all cases based on morphological and immunohistochemical aspects. All neoplasms exhibited strong anticytokeratin 7 immunostaining, as recommended for diagnosing endometrial adenocarcinoma in nonhuman primates [8].

An association between advancing age and neoplasia incidence has been well documented in several species, including nonhuman primates [23]. No cases of neoplastic proliferation were observed in young females in this study. This fact supports the idea that the incidence of neoplasms in the female reproductive system of nonhuman primates may be related to aging due to an increase in life expectancy in human care settings. Similarly, previous studies have described a possible correlation between increasing age and the occurrence of uterine leiomyomas in macaques [11, 22] and intestinal adenocarcinomas in rhesus monkeys ( *Macaca mulatta* ) [24]. Two nonmutually exclusive mechanisms can explain the association between neoplasia and aging: carcinogenesis occurs over time, and age‐related molecular changes may render older tissues more susceptible to environmental carcinogens [25].

## 5 Conclusions

Our findings suggest no significant differences in the prevalence of ovarian and uterine spontaneous neoplasm presentation in neotropical primates based on a translational approach. Ovarian neoplasms were all classified as lesions without clinical significance. All uterine neoplasms are malignant with metastatic potential. Despite the low prevalence, female reproductive neoplasms should be included as a potential cause of neotropical primate deaths.

We suggest periodic in vivo reproductive monitoring of adult female neotropical primates for early diagnosis and therapeutic intervention.

## Conflicts of Interest

The authors declare no conflicts of interest.

## Data Availability

The data that support the findings of this study are available from the corresponding author upon reasonable request.
